# Expression of Phi11 Gp07 Causes Filamentation in *Escherichia coli*

**DOI:** 10.2174/1874285801812010107

**Published:** 2018-04-30

**Authors:** Avijit Das, Sumit Biswas, Malabika Biswas

**Affiliations:** BITS Pilani, K.K.Birla Goa Campus, Zuarinagar, Goa-403726, India

**Keywords:** Filamentation, *E. coli*, Drug-resistant, Therapeutic strategies, Bacteriophage, Host transcription

## Abstract

**Background:**

The Gp07 protein of aureophage Phi11 exhibits growth inhibitory effects when overexpressed in *E. coli* .The protein harbors two domains- an amino terminal Bro-like domain and a carboxy terminal Ant superfamily like KilA domain, of which the KilA domain retains the growth inhibitory effect of Gp07.

**Methods:**

We studied the effects exerted by the overexpression of Gp07 and its separate domains upon the growth rate as well as the morphology of the *E. coli* cells. Additionally, we generated a mutant of Gp07 (designated as ΔGp07) by deleting the first eleven amino acid residues from the amino-terminal region of Gp07, and studied its growth inhibitory effects upon *E. coli.*

**Results:**

Our results indicate that Gp07, ΔGp07 as well as the Carboxy-terminal region of Gp07 upon overexpression, retards the growth rate of the *E. coli* cells and also induces filamentation in the cells. Surprisingly, our data clearly suggests that the growth inhibition and filamentation induced by the the amino-terminal domain of Gp07 is temporal in nature.

**Conclusion:**

The carboxy-terminal of domain of gp07 is essential for its activity.

## 1 INTRODUCTION

The growing incidence of drug-resistant pathogenic bacteria is a major concern today. It has now become critical to devise novel therapeutic strategies and antibacterial alternatives to combat infections caused by multi-drug resistant pathogens. Several bacteriophages have been reported to utilize the host machinery for their survival. Such bacteriophages code for certain proteins which alter some of the essential host proteins. These phage encoded proteins can either be bacteriostatic or bactericidal in nature [1]. Information about such phage encoded proteins can be instrumental in designing drugs against pathogenic bacteria [2].

Phi11 is a temperate bacteriophage (serogroup B [3] and lytic group III) which infects *Staphylococcus aureus*. It harbours a 43.6 kb [4] double stranded linear DNA genome (G+C content of 35-37%) [5]. Being a temperate phage, Phi11 can undergo both the lytic as well as the lysogenic mode of development. The sequence of Phi11 *gp07* revealed the presence of two conserved domains- the amino terminal domain belonging to baculovirus repeated ORF[s] Bro family and the carboxy terminal domain belonging to ANT superfamily which bears homology to KilA domain [6]. The Bro proteins were proposed to be DNA-binding in nature and are involved in the regulation of viral and host transcription, replication and chromatin structure [7, 8]. It has already been reported that bacteriophage lambda harbours a gene called the kil gene. Overexpression of the kil gene leads to inhibition of FtsZ ring formation, which in turn induces filamentation of *E. coli * [9].

To look into the effect of *gp07* gene upon the host cells, we have cloned and overexpressed the Phi11 Gp07, its domains, as well as a truncated Gp07 (ΔGp07) as histidine-tagged variants. ΔGp07 has been generated by deletion of 11 amino acid residues present at the amino terminal region of Gp07. Overexpression of the Gp07, ΔGp07 as well as the KilA-C domain exhibited an inhibitory effect upon the cell division of *E. coli*. The Bro domain upon overexpression did exhibit some degree of toxicity; however, the inhibitory effect exerted by Gp07, ΔGp07 and the KilA-C domain expression far exceeded that of the Bro domain. This study offers a firsthand preliminary report about the cell growth inhibitory role of Phi11 *gp07*.

## 2 MATERIALS AND METHODS

### 2.1 Bacterial Strains, Phage Strains, and Growth Conditions

Phi11 has been grown in our laboratory according to the method of Lee and Iandolo [10]. *S. aureus* RN4220 was grown at 37˚C in Trypticase soy broth [11]. *E. coli *BL21 (DE3) and *E. coli *XL1 Blue cells were grown at 37˚C in Luria broth [12]. Appropriate antibiotics were added to the growth media as required.

### 2.2 Molecular Biological Techniques

Isolation of Phi11 chromosomal DNA was carried out by some modification of prescribed method [10]. All DNA manipulations such as plasmid DNA isolation, polymerase chain reaction, restriction enzyme digestion of DNA, ligation of DNA fragments, transformation were carried out according to standard procedure [12]. Sequencing of all Phi11 DNA inserts (amplified by PCR) were performed in UDSC (New Delhi, India). Standard methods were employed to estimate the amount of protein; SDS-PAGE, polyacrylamide gel staining were also carried out by standard procedure [13].

### 2.3 Bioinformatics Analysis of Gp07

Genome databases in NCBI (http://www.ncbi.nlm.nih.gov/) and Pfam (http://pfam.sanger.ac.uk/) were used for bioinformatics analysis of Gp07. EMBOSS programs (https://www.ebi.ac.uk/Tools/emboss/) were used for different bioinformatics analyses like molecular weight determination, charge and presence of different amino acid. Sequence similarity search was carried out by BLAST server (http://www.blast.ncbi.nlm.nih.gov/blast/Blast.cgi). The search for Phi11 *gp07* gene was carried out using BLASTP in different databases in Caudovirales order and 50% or more identity was taken as the threshold [14]. Sequences similar to the *gp07* of Phi11 were detected from different non-redundant (NR) protein sequence databases by BLAST (blastp) program 2.2.32+. Alignment of full-length Gp07 protein was performed by Clustal Omega program 1.2.1 (http://www.ebi.ac.uk/Tools/msa/clustalo/). Using all the proteins which shared 50% identity to the Phi11 Gp07, a dendrogram was constructed using ClustalW2 Phylogeny program (https://www.ebi.ac.uk/Tools/phylogeny/clustalw2_phylogeny/) with neighbor-joining method.

### 2.4 Cloning of gp07, Its Domains and Truncated Gp07(ΔGp07)

To clone Phi11 *Gp07*, we employed a polymerase chain reaction with DreamTaq DNA Polymerase from ThermoFisher Scientific using primers C-Gp07-F and C-Gp07-R (Table **1**). The template used was Phi11 genomic DNA. The product obtained was 825bp in size and was cloned into pGEM-T Easy vector (Table **2**) according to the manufacturer’s protocol (www.promega.com/protocols/). The recombinant pGEM-T Easy vector was transformed into competent *E. coli *XL1 Blue cells. The recombinant pGEM-T Easy vector carrying no mutation in the gp07 (as confirmed by DNA sequencing) was chosen for further work. *gp07* was further subcloned into pET28a (Table **2**) and designated as Gp07. This cloning protocol has included ten extra amino acid residues (including six histidine residues) at the carboxy terminal end of the putative Gp07 protein.

Similarly ΔGp07 (generated by deletion of 11 amino acid residues present at the amino terminal region of Gp07), Bro-N(rNTD) and KilA-C(rCTD) domains were PCR amplified from Phi11 genomic DNA using the primers C-ΔGp07-F, C-gp07-R; C-gp07-F, C-Bro-R and N-KilAC-F, N-gp07-R respectively (Table **2**). The resulting PCR products of 792bp(ΔGp07), 420bp(rNTD) and 405bp(rCTD) were cloned into pGEM-T Easy vector (Table **1**), sequenced and further sub-cloned into pET28a (Table **1**). All the recombinant constructs were transformed into *E. coli *BL21(DE3) cells (Novagen, USA) and healthy transformants (carrying no mutation) were selected for overexpression.

### 2.5 Overexpression Assays of Gp07, Its Domains and ΔGp07 in *E. coli*

*E. coli *cells harbouring Gp07, rNTD, rCTD and ΔGp07 were separately grown in LB (with 50µg/ml kanamycin), overnight at 37˚C with shaking. At ~12-14 h, these cultures were subcultured to OD_600_ of ~0.05 in LB (with 50µg/ml kanamycin) followed by induction with 0.5mM IPTG. Following induction, OD_600_ of the cultures were measured at 30 minutes. This was followed by OD_600_ measurements every 1 hour for 8 hours. The entire procedure was repeated three times and the mean values and the standard errors were calculated. The CI repressor protein of Phi11 [15] was used as a negative control.

### 2.6 Examination of cell morphology of *E. coli* (Harbouring Gp07, rNTD, rCTD or ΔGp07) Using Phase-Contrast and Fluorescence Microscopy

Following induction with IPTG, cells overexpressing either Gp07, rNTD, rCTD or ΔGp07 (were harvested at two different time point (5 h and 8 h) and washed with 0.9% sterile NaCl solution. The washed cells were resuspended in saline, spread on a clean glass slide, air dried at room temperature and then fixed with methanol for 5 minutes at room temperature. The fixed cells were thoroughly washed with sterile distilled water, dried at room temperature, and then 10µl of poly-L-lysine (5 µg/ml of distilled water) was spread over the sample (16).

To study the nucleoid structures formed in the *E. coli *cells (expressing either Gp07, rNTD, rCTD, ΔGp07 or none), the desired samples were stained with DAPI solution [2-(4-Amidinophenyl)-6-indolecarbamidine dihydrochloride, 4′, 6-Diamidino-2-phenylindole dihydrochloride] (Sigma-Aldrich Chemicals Pvt Limited) (10ul; 5ug/ml of saline), which binds specifically to DNA [16]. The cells were observed through an Olympus IX51 inverted microscope oil immersion objective (100X), combining the phase-contrast system and the fluorescence system (a U-LH100HG apparatus) in a dark room. When the light of a halogen lamp was reduced to an appropriate level, the fluorescent nucleoid structures and cell shape were clearly visible at the same time. The microscope was also equipped with a ProgRes® CT3 camera (Jenoptik, USA). Photographs were captured and analyzed by ProgRes^®^ CapturePro 2.8.8 software. The CI repressor protein of Phi11 was used as a negative control.

### 2.7 Examination of *E. coli* Cell Morphology with Scanning Electron Microscopy (SEM)

To prepare samples for scanning electron microscopy, overnight cultures of *E. coli *cells carrying gp07, its domains and ΔGp07 (expressing either Gp07, rNTD, rCTD or ΔGp07) were separately allowed to grow to OD_600_ 0.05 in fresh LB at 37°C. Wild type *E. coli *as well as *E. coli *overexpressing Phi11 CI were used as controls. The cells were induced with 0.5 mM IPTG and cultured at 37°C, 120 rpm shaking for another 8 h. Cells were then harvested by centrifugation for 10 min at 7500 g followed by washing with 1X PBS (pH 7) twice. The final cell pellets were re-suspended in appropriate volume of 1X PBS and thinly smeared on clean cover-slips. The prepared cover-slips were kept for air drying. The dried cover slips were then washed three times with 1X PBS and the cells were pre-fixed with 3% glutaraldehyde (in 1X PBS) for 1hr at room temperature. The excess glutaraldehyde was removed by washing the cells, three times, with 1X PBS. The cells were then treated with 1% OsO_4_ at 4°C, and left overnight. Following this, the cells were again washed three times with 1X PBS at room temperature. Cells were then dehydrated using a series of ethanol solutions (10%, 30%, 50%, 70% and 90%, 5 min each) and finally transferred to 100% ethanol for 10min. To completely dehydrate the cells the prepared slides were transferred to a CPD chamber (Leica EM CPD300). The specimens were placed onto foil which was glued onto a metal specimen holder after the CPD completion. The specimens were coated by gold-palladium mixture at 5nm thickness using Leica EM ACE200. The SEM images of cells were obtained using a FEI Quanta FEG 250 Scanning electron microscope.

## 3 RESULT

### 3.1 Pfam Analysis of Gp07

Pfam (http://pfam.xfam.org) was used for full length protein sequence analyses. In Phi11 genome, amino acid sequence analysis by Pfam indicated that the locus tag “phi11_07” [ORF7, which was predicted as a Gp07] exists adjacent to the Cro-repressor (ORF6) [17]. The Gp07 protein consists of two conserved domains, an amino terminal domain (NTD) and the carboxy terminal domain (CTD). Out of the 274 amino acids of Gp07, the NTD ranges from 25th to 118th amino acids and belongs to baculovirus repeated ORF(s) Bro-N family (6). The CTD ranges from 160th to 263rd amino acid residues of Gp07 and belongs to KilA-C domain under ANT superfamily Fig. (**1A**). KilA-C was initially reported as the carboxy terminal domain of phage PI KilA (7). According to Pfam analysis, the E-value for Phi11 Bro-N is 1.5e-27 and 5.4e-30 for KilA-C domain. Bro gene (5 members) has been reported in Bombyx mori nucleopolyhedrovirus (BmNPV), a double stranded virus infecting lepidopterans (6). Further, there are reports of the existence of bro gene homologues in transposons, bacteriophages and probacteriophages [8, 18, 19]. The Bro proteins are regulatory proteins involved in regulating transcription, replication in certain viruses and their host organisms.

### 3.2 Gp07 of Phi11 Belongs to the Caudovirales

**Fig. (2):**
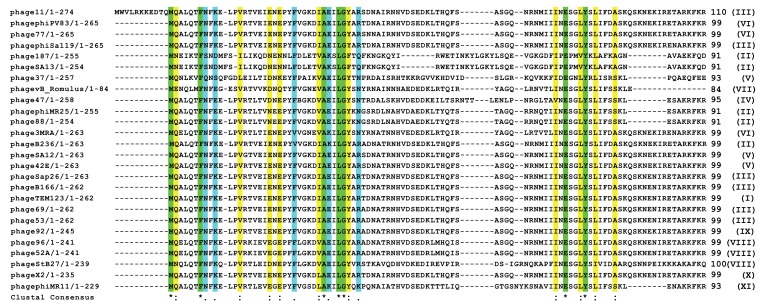
**Sequence analysis of the full-length protein of some phages in Caudovirales.** Sequences are designated by their phage name and followed by protein length. Phi11 Gp07 has been aligned with ClustalO (See Materials and Methods for details). The coloring reflects the conservation profile at clustal consensus of amino acids. ‘*’ identical residues (M,F,A,L,G,E,Y), all shaded green. ‘ : ’ highly conserved residues (Q,V,I,E,N,D,Y,F) shaded yellow. ‘ . ’ weakly conserved residues (S,D,E,F,V,R,Q,N,K) shaded turquoise. Phage Phi11 unique residues region (MWVLRKKEDTQ) all coloured gray.

According to International Committee on Taxonomy of Viruses (ICTV) classification, Caudovirales (the bacteriophages that have tails) is a taxonomic order within the kingdom Virus. There are at least 1442 phages in this order with complete genome sequence known to us. By BLASTP search of non-redundant (NR) databases in Caudovirales order, it was observed that apart from the conserved residues, the first eleven amino acids of Phi11 Gp07 protein are extremely unique Fig. (**1A**). These eleven amino acid residues are absent in all other proteins harbouring KilA-C and Bro-N domains, in Caudovirales order as well as outside the Caudovirales order Fig. (**2**). All this twenty-six phages (vide Fig. **2**) belong to the Myoviridae family of viral systems. The neighbor-joining phylogenetic analyses revealed that the phage most closely related evolutionarily to phage Phi11 is *Staphylococcal* phage 42E Fig. (**1B**). It was reported that bro-like gene family is widespread among large double-stranded DNA viruses of invertebrates and bacteria [8]. Additionally, other reports revealed shuffling of domains which leads to different patterns of gene organizations [7].

### 3.3 Inhibition of Cell Growth by Expression of Gp07, Its Domains and ΔGp07

**Fig. (3):**
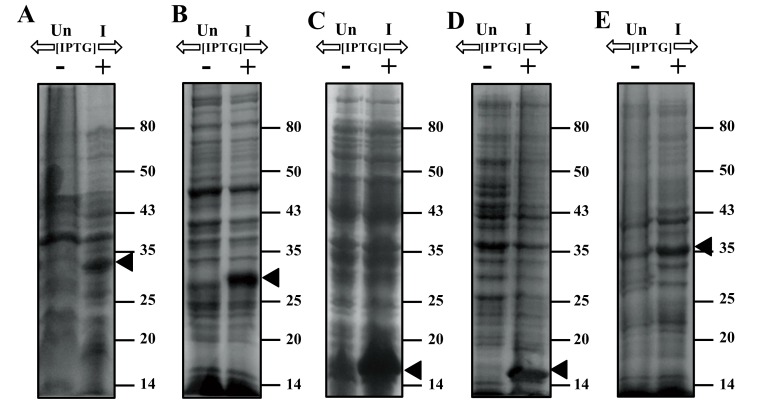
**Overexpression of Gp07, its domains (rNTD, rCTD), Δgp07 and a control protein in *E. coli*.** Overexpression of the recombinant proteins (induced with 0.5mM IPTG for 2hrs at 37^o^C) were analysed on SDS-12%-PAGE. (A) Overexpression of Gp07 (B) Overexpression of ΔGp07 (C) Overexpression of rNTD; (D) Overexpression of rCTD; (E) Overexpression of control protein. Arrowheads indicate the overexpressed protein. **Un** indicates uninduced cellular extract; **I** indicates induced cellular extract.

*E. coli *cells harbouring either Gp07, rCTD or ΔGp07 (overexpressions were confirmed by SDS-12% PAGE, Fig. **3**), upon induction by 0.5mM IPTG at 37°C for 5 h, showed a rapid inhibition in growth rate Fig. (**4**). This data is indicative of the growth inhibitory nature of Gp07. However, it was observed that there was a minor increase in OD_600_ of the cells after approximately 6 hours of induction of Gp07, rCTD or ΔGp07 Fig. (**4**). On the other hand, *E. coli *cells harbouring rNTD (overexpression was confirmed by SDS-12% PAGE, Fig. **3**) upon induction by 0.5mM IPTG at 37˚C for 3 h also showed inhibition in growth rate. However, in this case, the growth inhibition was much lower than that of Gp07, rCTD or ΔGp07 Fig. (**4**). More interestingly, in this case, resumption of growth by the induced cells occurred earlier than that in case of Gp07, rCTD or ΔGp07 and there was a substantial increase in the optical density (OD_600_) at the end of 5 h. In case of the negative control, CI, there was no significant change in the OD of the overexpressing cells as compared to the wild type cells.

### 3.4 Microscopic Observation of *E. Coli *Cells Upon Overexpression of Gp07, Its Domains and ΔGp07

*E. coli *cells (harbouring no plasmid) and *E. coli *cells harbouring Gp07, induced with 0.5mM IPTG were examined under phase contrast microscope as well as SEM. It was observed that, the host cells carrying Gp07 upon induction became filamentous Figs. (**5E** , **5G** and **6C**). Moreover, irregular multiple nucleoid structures could be observed upon staining of the induced cells with DAPI Figs. (**5F** and **5H**). This suggests that normal DNA replication progressed in the cells. The filamentation was not reversible in case of *E. coli *cells overexpressing Gp07 Figs. (**5E** , **5G** and **6C**). Similar results were also observed in case of *E. coli *cells harbouring rCTD and ΔGp07 Fig. (**5**). In case of rNTD however, the filamentation was reversed at the end of 8 h as is evident from Figs. (**I**/**5K** and **6D**). Examination of the induced *E. coli *cells harbouring rNTD by phase contrast microscopy as well as SEM showed an increased number of *E. coli *cells with normal morphology at the end of 8 h Figs. (**5K** and **6D**). This further strengthens our observation that compared to rNTD domain, the rCTD domain is essential for the growth inhibitory effect of Gp07. In its absence, the rNTD alone cannot retain this activity of Gp07. The negative control, CI, had no effect on the morphology of the host cells Figs. (**5C**, **5D** and **6B**).

## 4 DISCUSSION

Our preliminary work showed that overexpression of Gp07, ΔGp07, as well as its domain KilA-C (rCTD) significantly inhibited the growth of *E. coli *following induction with IPTG. Similar observations have already been reported in host cells expressing the icd gene of P1 [20], whereby, *E. coli *cells expressing the Icd protein of bacteriophage P1, become filamentous in nature. There have also been reports that the Rac prophage (21) of *E. coli *harbours a gene called kil which codes for a 73 amino acid protein. The overexpression of this kil gene was shown to block cell division resulting in filamentation of the host cell. It has further been reported that this inhibition is relieved by excess FtsZ [21]. Studies on the Kim (Qin) prophage [22] also indicate the inhibition of divisome formation in *E. coli*. The kil gene of bacteriophage lambda has also been reported to induce filamentation in host cells [9]. Haeusser et al., have already reported that cell division in *E. coli *host cells are blocked by the Kil protein of bacteriophage λ. The Kil protein possibly ablates the FtsZ rings in *E. coli*, thereby blocking cell division. The block can however be inhibited by an abundance of FtsZ [9].

Our other observation in *E. coli *cells overexpressing Gp07, ΔGp07 as well as its domain KilA-C (rCTD), was the very slight increase in the optical density of the cells measured at 600 nm from 5-6 h following induction. We initially assumed that the growth inhibitory effect exerted by Gp07 might be temporal in nature and hence the slight increase in OD; however, cell morphology studies indicated that the cells upon Gp07, ΔGp07 and KilA-C expression became filamentous in nature, which is an indication of inhibition of cell division. This slight increase in the OD value can be attributed to the increase in the length of the filamentous cells and not necessarily due to the resumption of cell growth. Further, CFU analysis of *E. coli *cells indicated that there was no significant increase in the number of CFU after overexpression of Gp07 in *E. coli*, at different time intervals (data not shown).

Analysis of the amino acid sequence of Gp07 further indicates that the first eleven amino acids of Phi11 Gp07 protein are extremely unique Figs. (**1A** and **2**). To check if the eleven amino acid residues had any role in the growth inhibitory effect of Gp07, we deleted these amino acids and generated the mutant, ΔGp07. It is interesting to note that ΔGp07 retained the growth inhibitory effect displayed by full- length Gp07. Thus we can assume that the first eleven amino acids of Phi11 Gp07 protein do not play any role in its growth inhibitory effect. Further bioinformatics analysis of Gp07 showed that the two identified domains, Bro-N and KilA-C are widely spread and present in different organisms. Considering our cell growth kinetics experiments, studies on cell morphology with phase-contrast and fluorescence microscopy as well as SEM data of Gp07 and its domains, it is clear that the carboxy terminal domain of Gp07 (KilA-C domain) is more important for its growth inhibitory function in *E. coli*. In case of the Bro-N domain, the data clearly suggests that the growth inhibition induced by the Bro-N domain alone, is temporal in nature.

## CONCLUSION

Taken together, our results indicate three things: i. Gp07 and ΔGp07 exert a growth inhibitory effect upon *E. coli *cells, ii. The KilA-C domain of Gp07 is essential for its activity, and iii. The Bro-N domain is not essential for the growth inhibitory effect; moreover, the growth inhibition of *E. coli *induced by the Bro domain is temporal in nature.

## ETHICS APPROVAL AND CONSENT TO PARTICIPATE

Not applicable.

## HUMAN AND ANIMAL RIGHTS

No Animals/Humans were used for studies that are base of this research.

## CONSENT FOR PUBLICATION

Not applicable.

## Figures and Tables

**Fig. (1):**
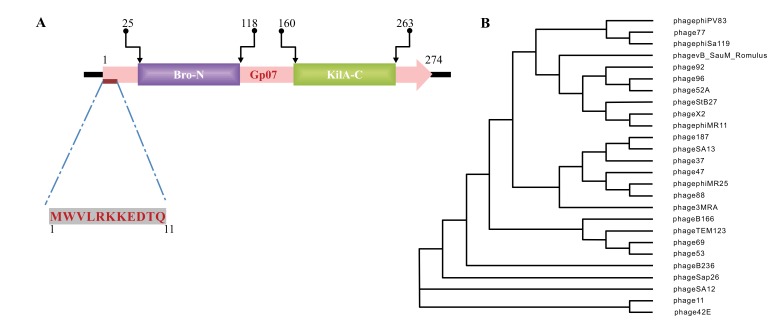
**Domain organization of Gp07.** (A) Pfam analysis indicates that Gp07 harbours two domains- the amino terminal Bro domain and carboxy terminal KilA domain; the first eleven unique amino acids are coloured gray. (B) Phylogenetic analysis of Phi11 Gp07 has been aligned with ClustalO (See Materials and Methods for details) by neighbour-joining method. The Phi11 is evolutionary closely related with phage 42E.

**Fig. (4):**
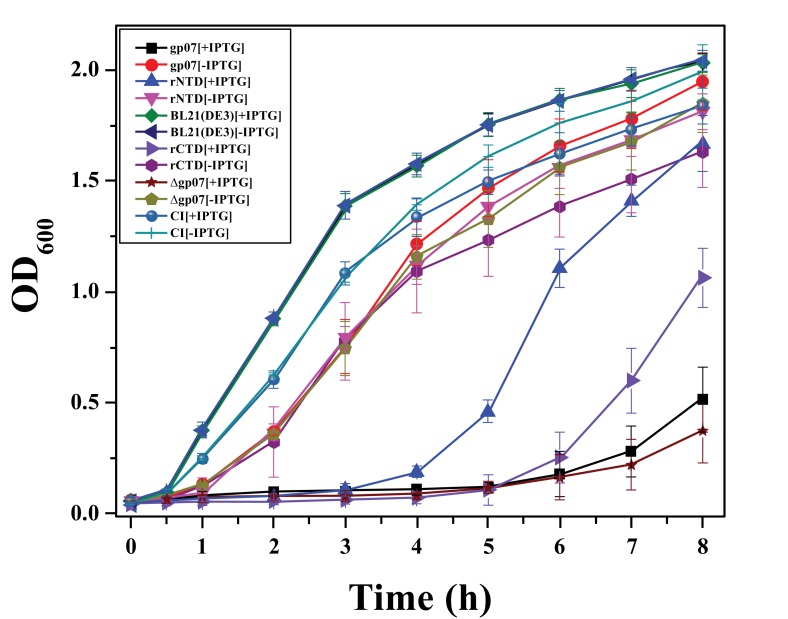
**Effect of Gp07, its domains and ΔGp07 upon the growth of *E. coli*.** Overexpression of Phi11 Gp07, rCTD as well as the ΔGp07 inhibited growth of the host cells. Overexpression of rNTD initially repressed host cell growth but resumption of cell growth was seen after a few hours. The overexpressed control protein (CI) had no effect upon the growth of *E. coli *cells. All experiments were performed in triplicate. The standard error of the mean was used to calculate the error bars. In case of those points where the experimental variations are too small, the error bars are not visible.

**Fig. (5):**
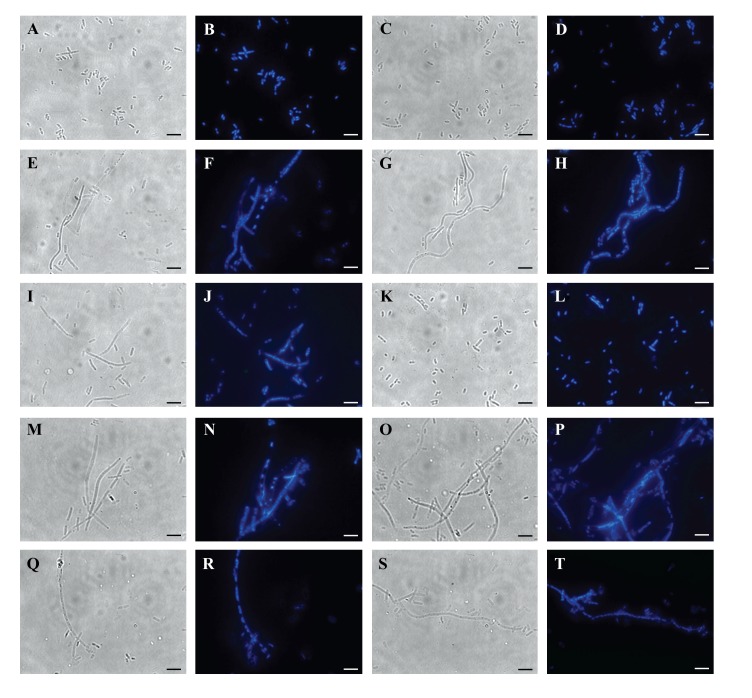
**Effect of overexpression of Gp07, its domains and ΔGp07 on the morphology of host cell.** (A) Morphology of wild type host cells (*E. coli*) under microscope at 100X magnification. (C) Morphology of *E. coli *cells overexpressing the control protein. (E,I,M,Q) Morphology of *E. coli *cells overexpressing Gp07, rNTD, rCTD and ΔGp07 respectively at 5h, as observed by phase contrast microscopy. (G,K,O,S) Morphology of *E. coli *cells overexpressing Gp07, rNTD, rCTD and ΔGp07 respectively at 8h, as observed by phase contrast microscopy. (B) DAPI stained normal host cells (*E. coli *BL21 DE3 not carrying any recombinant DNA) under fluorescence microscope. (D) DAPI stained E.coli cells overexpressing the control protein. (F,J,N,R) DAPI stained E.coli cells overexpressing Gp07, rNTD, rCTD and ΔGp07 respectively at 5h, under fluorescence microscope. (H,L,P,T) DAPI stained *E. coli *cells overexpressing Gp07, rNTD, rCTD and ΔGp07 respectively at 8h, under fluorescence microscope. All the scale bars are 5µm.

**Fig. (6):**
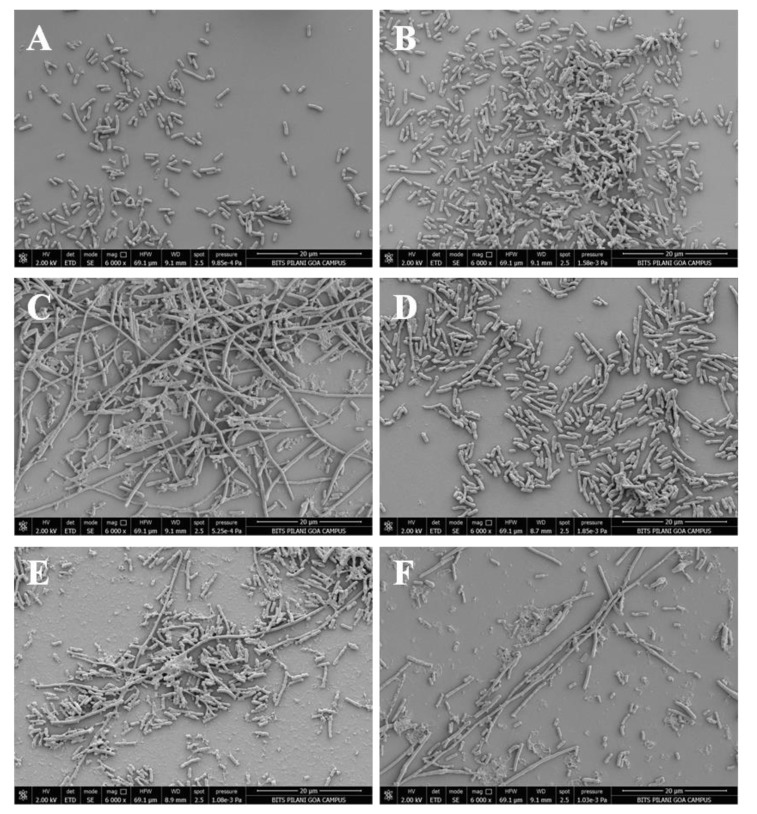
**Scanning electron micrographs of Wild type (WT), Control protein, Gp07, rNTD, rCTD and ΔGp07 expression in *E. coli*.** (A) Wild type (WT), (B) Control protein (C) Gp07, (D) rNTD, (E) rCTD, (F) ΔGp07; all scale bars are 20 µm.

**Table 1:** Primer used in this study to express the Gp07, rNTD, rCTD and ΔGp07.

**Name**	**Sequence(5′-3′)^#^**	**Purpose**
**C-gp07-F**	AAACCATGG**GAATGTGGGTGTTGAGGAAAAAGGAGG**	Forward primer for synthesis of Gp07 and rNTD
**C-gp07-R**	AAACTCGAG**CGCTCCCCCTAAATTAGCTTCATAAC**	Reverse primer for Synthesis of Gp07 and **Δ**Gp07
**C-Bro-R**	AAACTCGAG**GTCTGGATCTTTTAATGTTTGTTCAATTACATTG**	Reverse primer for the synthesis of rNTD
**N-KilAC-F**	AAACATATG**TACATCATTACAGTGTTGACTGAGTATAAGAAAG**	Forward primer for the synthesis of rCTD
**N-gp07-R**	AAACTCGAG**TTACGCTCCCCCTAAATTAGCTTCATAACC**	Reverse primer for the synthesis of rCTD
**C-Δgp07-F**	AAACCATGG**AAATGCAAGCATTACAAACATTTAATTTTAAAGAGC**	Forward primer for synthesis of **Δ**Gp07
**#**Used primer, restriction sites are underlined; for PCR, **T_m_** is calculated for the bases in bold.		

**Table 2:** Plasmid used in this study and their derivatives.

**Plasmids**	**Source**	**Description**
pGEM-T Easy	Promega	*amp*^r^, *lacZ*, cloning vector
pET28a	Novagen	*kan*^r^, T7 lac, His-tag, expression vector
pGp07-T	This study	gp07 cloned in pGEM-T Easy vector
pGp07	This study	gp07 cloned in pET28a
prNTD-T	This study	rNTD cloned in pGEM-T Easy vector
prNTD	This study	rNTD domain cloned in pET28a
prCTD-T	This study	rCTD domain cloned in pGEM-T Easy vector
prCTD	This study	rCTD domain cloned in pET28a
pΔGp07-T	This study	ΔGp07 cloned in pGEM-T Easy vector
pΔGp07	This study	ΔGp07 domain cloned in pET28a
